# Number and Grammatical Gender Attraction in Spanish Pronouns: Evidence for a Syntactic Route to Their Features

**DOI:** 10.5334/joc.416

**Published:** 2025-01-07

**Authors:** Margaret Kandel, Claudia Pañeda, Nasimeh Bahmanian, Mercedes Martinez Bruera, Colin Phillips, Sol Lago

**Affiliations:** 1 Harvard University, Cambridge, US; 2 University of Oviedo, ES; 3 Open University of Catalonia, ES; 4 Goethe University, Frankfurt, DE; 5 University of Oxford, UK; 6 University of Maryland, College Park, US

**Keywords:** pronouns, production, agreement attraction, number, grammatical gender, Spanish

## Abstract

When a speaker produces a pronoun, they must choose a form that carries the appropriate features. The current study investigates how speakers identify these features. We consider two possible routes: a conceptual-lexical route, whereby pronouns derive their features from the concept of the referent, and a syntactic route, whereby pronoun form is determined through a feature matching operation with the linguistic antecedent. We hypothesize that the use of these two routes should be differentially susceptible to interference from representations other than the pronoun’s referent. We use agreement attraction to distinguish them. In two experiments, we test whether Spanish speakers produce number and grammatical gender attraction errors. We observe small but reliable attraction effects for both features, demonstrating that pronoun formulation can be disrupted by the linguistic representations of nearby nouns. These attraction effects suggest that speakers can use a syntactic route to pronoun form.

## Introduction

An essential part of language production is transforming preverbal concepts into words. Production models propose that concepts are grammatically encoded as lemmas, which are then phonologically encoded as lexemes and finally articulated (Bock & Levelt 1994; Levelt 1989). For example, the concept of multiple exemplars of a certain type of fruit may be grammatically encoded using the lemma *apple* with a plural number feature, phonologically encoded as the lexeme /æplz/, and articulated as [ˈæplz].

However, concepts that can be grammatically encoded as nouns may at times appear as pronouns instead. For example, if the concept ‘multiple apples’ is contextually salient, in focus in the discourse (Schmitt, Meyer & Levelt 1999), or has recently been mentioned within a sentence, a speaker may decide to refer to it as *they* rather than as *apples*, as in *Those apples are riper than the pears, aren’t they?*.

Although much research has tried to determine when a speaker decides to refer to a concept using a pronoun (see Arnold & Zerkle 2019 for review), it is an open question how speakers determine the appropriate pronoun form to produce. For instance, do speakers consult conceptual and/or linguistic representations when determining pronoun form? How do speakers access the features of the relevant representations? Can non-antecedent noun phrases influence this process? Our study uses agreement attraction to investigate how speakers determine pronoun form in sentences where a pronoun is coreferential with a linguistic antecedent (e.g., the determiner phrase *those apples* in the example above). We consider two potential routes: a *conceptual-lexical route*, whereby pronoun form is chosen independently of a linguistic antecedent, and a *syntactic route*, whereby pronoun form is established through a matching operation with an antecedent, similar to subject–verb agreement. These routes make different predictions about whether or not other non-antecedent noun phrases can influence pronominalization and thus whether one should expect agreement attraction effects.

### Two routes to determine pronoun form

**Figure 1:**
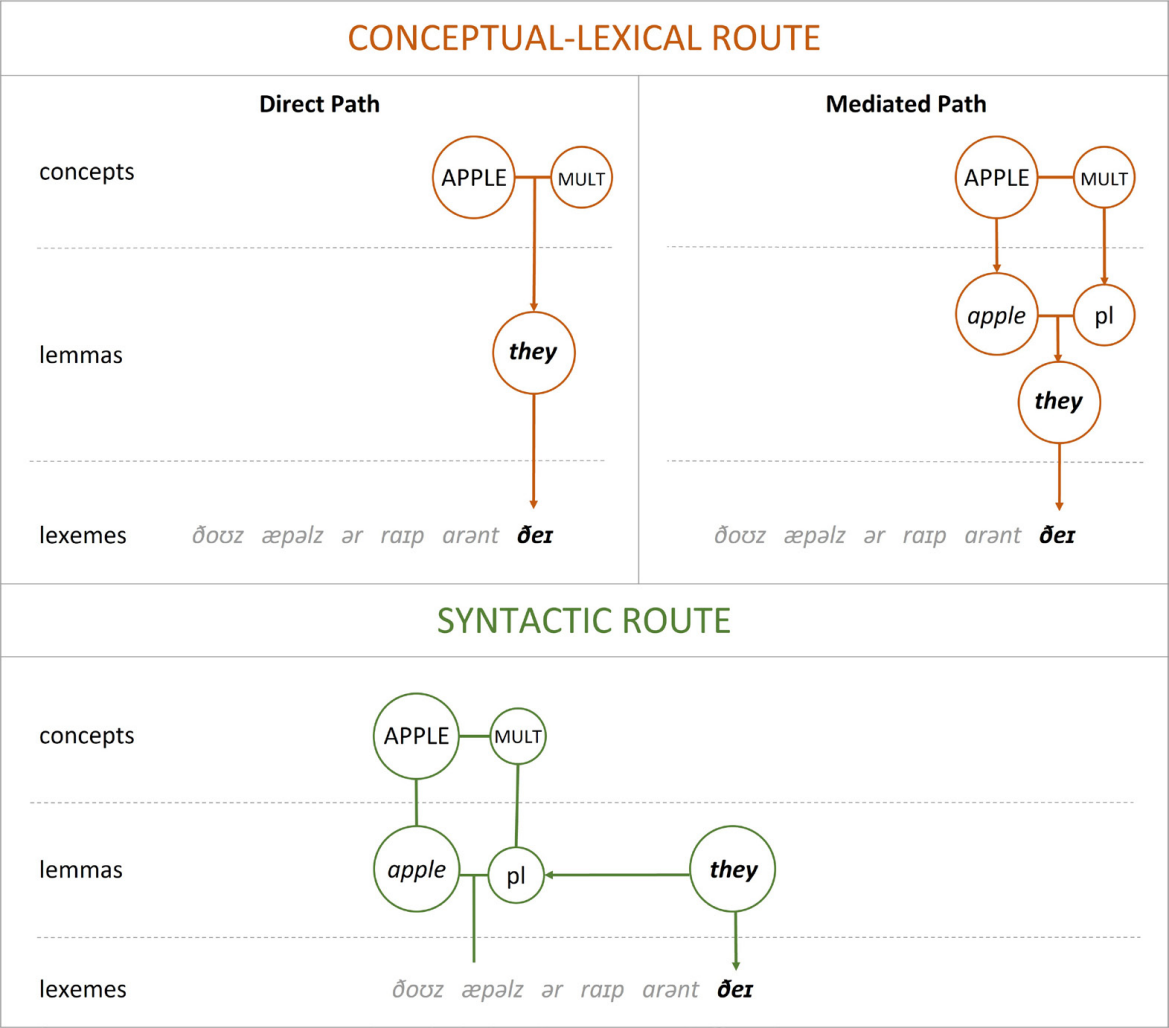
Summary of the two routes to determine pronoun form in production. *Note*. The figure demonstrates the two routes for selecting the pronoun *they* in the sentence *Those apples are ripe, aren’t they?*. Abbreviations: “MULT” denotes the concept of multiplicity, and “pl” denotes a grammatical plural feature. In the conceptual-lexical route, the concepts APPLE and MULT provide the features for the pronoun “they”, either directly (Direct Path) or through activating the corresponding representations at the lemma level (Mediated Path). In the syntactic route, the features for the pronoun “they” are accessed from the lemma-level representations corresponding to the linguistic antecedent “apples”.

In the conceptual-lexical route (referred to by Meyer & Bock 1999 as the “conceptual hypothesis”) the speaker determines pronoun form in a similar way to noun form: by activating a concept like ‘multiple apples’, which is then grammatically encoded as a lemma and phonologically encoded as a lexeme. The lemma that serves as the input for the phonological encoding stage would just be a pronoun rather than a noun. Within the conceptual-lexical route, two paths are potentially available to determine pronoun form (Figure 1). On the direct path, the pronoun’s lemma is accessed directly from the concept. This may be possible when all the features that determine pronoun form have conceptual correlates. For example, a speaker may refer to some apples with the plural pronoun *they* because the concept has a multiplicity feature. Alternatively, on the mediated path, the pronoun form is accessed from the concept via an associated noun in the mental lexicon (Jescheniak, Schriefers & Hantsch 2001; Meyer & Bock 1999; Schmitt, Meyer & Levelt 1999). In this case, the concept ‘multiple apples’ activates the lemma *apple* with a plural number feature, which in turn activates the lemma for the pronoun. This form of mediation may be required when pronoun form is influenced by grammatical features without conceptual correlates, such as grammatical gender. For example, in Spanish, the pronoun referring to a set of apples is not only plural, but also feminine, as in ¿*Te las vas a comer?* (‘Are you going to eat them._FEM_?’). The pronoun *las* reflects the feminine gender of the noun *manzana* (‘apple’), which cannot be attributed to apples being conceptually female.

The conceptual-lexical route may be the only one available when pronouns are used deictically, without a linguistic antecedent, as when a speaker points to some apples and says, *They are ripe*. However, it may also apply when there is a linguistic antecedent, as in the sentence *Those apples are ripe, aren’t they?*, where the pronoun *they* co-refers with the antecedent *those apples*. In this case, the pronoun and antecedent would be encoded fully separately: the concept of ‘multiple apples’ would first be grammaticality encoded as *apples* — and phonologically encoded as /æplz/ — and later in the sentence, the concept would be grammatically encoded as *they* — and phonologically encoded as /ðeɪ/. In this case, the antecedent and the pronoun end up sharing grammatical and/or morpho-phonological features as a collateral effect of encoding the same concept.

In contrast, in the syntactic route (similar to Meyer & Bock’s 1999 “lexical hypothesis”), a pronoun receives its features from the antecedent rather than the concept, similar to how verbs receive their features from a sentence’s subject phrase (Figure 1). Under this route, speakers access what has been previously said in the sentence or discourse and perform a matching operation between the antecedent and the pronoun’s features. The syntactic route may be important in cases when a concept has more than one possible grammatical encoding. For instance, the concept ‘scissors’ may be referred to using the plural noun *scissors* or the singular noun phrase *pair of scissors*. In this case, how a concept has previously been referred to in a discourse is important for selecting the correct pronoun form — *they* or *it*. This route to pronoun form selection can also capture grammatical gender agreement, since the linguistic antecedent has grammatical gender. The syntactic route may be readily applied in cases of intra-sentential pronominalization, when speakers could already be monitoring the content of the sentence in order to abide by constraints on sentence formulation, such as binding constraints and avoidance of noun phrase repetition.

To summarize, a key difference between the two routes is whether pronoun form is influenced by other parts of the sentence or previous discourse (syntactic route) or not (conceptual-lexical route). Our study assesses whether the syntactic route is used during the production of pronouns with intra-sentential antecedents in Spanish, a language in which pronouns mark the number and grammatical gender of their referent. We address these questions by investigating whether pronouns are subject to agreement attraction.

### Agreement attraction as a tool to arbitrate between the two routes

Observing what speakers say is usually not enough to arbitrate between the conceptual-lexical route and the syntactic route: If everything goes well during production, a pronoun’s form will be the same regardless of the route taken. However, speakers sometimes make errors, and these errors can help reveal which route was taken. Here we focus on *agreement attraction* errors, as in (1), where the pronoun *they* does not agree with the agreement controller *actor* but rather with the local attractor noun *soap operas*. In production studies, attraction effects are indexed by a higher number of agreement errors when the agreement controller and attractor have different features (e.g., *the actor in the soap operas*) than when they have the same features (e.g., *the actor in the soap opera*) (Bock, Nicol & Cutting 1999; Bock, Eberhard & Cutting 2004; Bock et al. 2006; Kandel & Phillips 2022; Kandel, Wyatt & Phillips 2024).

(1) *The actor in the **soap operas** rehearsed, didn’t they?

The finding that pronouns are subject to agreement attraction demonstrates that pronoun form can be influenced by elements that are neither the concept referred to by the pronoun nor its associated lemma. This finding is unexpected under the conceptual-lexical route but could arise naturally from the syntactic route, as retrieving an antecedent for feature-matching opens up an opportunity for other nouns in the sentence to interfere.

However, the interpretation that the syntactic route is supported by attraction phenomena crucially depends on the assumption that errors are driven by the presence of an attractor noun within the sentence. This is not obviously the case in most previous studies on pronoun production, which focus on number attraction (Bock, Nicol & Cutting 1999; Bock, Eberhard & Cutting 2004; Bock et al., 2006; Kandel & Phillips 2022; Kandel, Wyatt & Phillips 2024). Since grammatical number is usually associated with conceptual number (also referred to as notional number), in a sentence like (1), it is not necessary for attraction to come from the grammatical features of the attractor noun *soap operas* itself; rather, attraction may derive from the message-level representation of the plural concept encoded by this noun. For instance, *soap operas* should activate a conceptual representation of ‘multiplicity’ that could be erroneously combined with the concept ‘actor’, explaining why the pronoun appears as *they*. Indeed, pronouns are sensitive to conceptual number, leading speakers to produce plural pronouns to reference antecedents that are conceptually plural but grammatically singular (e.g., collectives such as *fleet*; Bock et al. 1999). Importantly, the conceptual properties of attractors, such as natural gender, have also been found to influence pronoun form (Slevc, Lane & Ferreira 2007). Thus, while number attraction indicates that pronoun form can be influenced by something other than the underlying concept or lemma, ruling out the conceptual-lexical route, it does not unequivocally support that the influence comes from a linguistic element, as required by the syntactic route.

One way to assess whether pronoun attraction can indeed derive from a linguistic element is to test whether pronouns show attraction from the grammatical gender of inanimate nouns, e.g., *manzana*._FEMININE_ in Spanish. Since grammatical gender doesn’t have systematic conceptual correlates (though see Boroditsky, Schmidt & Phillips 2003 and references therein for a different view), grammatical gender attraction cannot be attributed to the influence of the natural gender of other concepts in the message. Rather, grammatical gender attraction would demonstrate that pronoun form can be influenced by other parts of the sentence or previous discourse, as predicted by the syntactic route.

In support of this hypothesis, Meyer and Bock (1999) observed evidence of grammatical gender attraction with Dutch pronouns using a preamble completion paradigm. In this paradigm, participants heard a sentence containing two nouns (e.g., *aardappel*._COMMON_ ‘potato’; *badpak*._NEUTER_ ‘swimsuit’) and were shown a predicate that only matched one of them (e.g., *gaar* ‘cooked’). Participants were instructed to repeat the preamble sentence and add a continuation sentence with the structure *[pronoun] is [predicate]*, which required using a gender-marked pronoun (e.g., *Die*._COMMON_ *is gaar*). The results showed more pronoun form errors when the two nouns in the preamble mismatched in gender than when they matched, consistent with gender attraction.

While the presence of gender attraction in Meyer and Bock’s (1999) study provides some evidence in favor of a syntactic route, the observed effects may have in part arisen as a consequence of the elicitation paradigm used. This paradigm differs from natural production in ways that could inflate errors and/or favor the use of the syntactic route over the conceptual-lexical route. First, participants must interpret the grammatically-encoded representations of *both* nouns in the preamble sentence (the antecedent and the attractor) in order to determine which option should be pronominalized. Requiring speakers to consult the attractor as part of the pronominalization process may have artificially raised error rates relative to natural speech when the attractor would be irrelevant to pronoun form. In addition, the paradigm involves processes related to both parsing and working memory, as participants must remember, interpret, and correctly recall the provided preamble in order to repeat it and produce a continuation. This process might lead participants to plan constituents at a different time than they normally would if they are initially focused on recalling and repeating the preamble (Kandel & Phillips 2022). Also, elicited errors could reflect misinterpretations of the preamble structure rather than an influence of the attractor noun (Ryskin et al. 2021). Crucially, in Meyer and Bock’s (1999) task, speakers did not generate a prelinguistic message that they later transformed into linguistic output. Rather, the content of the message was predetermined by the provided preamble sentence and predicate, and the majority of the linguistic structure was already provided to participants — the only part of the utterance that speakers needed to plan for themselves was the pronoun. This may have led speakers to rely less on the message-level representations of the utterances to be produced, making them more likely to use a syntactic route than a conceptual-lexical route to determine pronoun form.

Concerns about the preamble paradigm have led some researchers to adopt description tasks to elicit agreement attraction effects (see Kandel & Phillips 2022; Kandel, Wyatt & Phillips 2024 for pronoun attraction; see Kandel & Phillips 2022; Kandel, Wyatt & Phillips 2022; Nozari & Omaki 2022; Veenstra, Acheson & Meyer 2014 for verb attraction). Scene description tasks alleviate some of the concerns of the preamble paradigm that might affect pronoun production: These tasks do not provide participants with any pre-packaged linguistic material to interpret or recall, speakers generate a message themselves based on the events of the scene, and they do not need to reference the attractor noun when deciding to pronominalize. Because of this, the salience of the message-level and linguistic-level representations of the antecedent and attractor noun are likely more comparable to natural speech production than in a preamble completion task, and speakers may be less biased to use the syntactic route. In line with this, two previous studies that used a description task to test number attraction in pronouns (Kandel & Phillips 2022; Kandel, Wyatt & Phillips 2024) obtained a smaller attraction effect than observed in previous preamble studies (Bock et al. 2006; Bock, Nicol & Cutting 1999; Bock, Eberhard & Cutting 2004), in which errors more closely resembled the attraction rates observed for verbs. This is consistent with the possibility that description tasks are less likely to lead to elevated errors and/or artificially favor using the syntactic route, particularly to the extent that this involves a matching process similar to subject–verb agreement.

However, prior scene description studies only elicited pronouns in one language (English) and only tested number attraction. Therefore, more research is needed to understand how pronoun form is determined cross-linguistically and from what source pronoun attraction errors arise, which provides insight into the pronoun planning route used. First, it is unclear whether the number attraction effect would replicate in other languages, particularly in languages like Spanish or Italian, whose speakers are known to be more sensitive to conceptual number during subject–verb agreement compared to speakers of languages with more limited inflectional morphology like English (Vigliocco 1996; Vigliocco, Butterworth & Garrett 1996; Vigliocco, Butterworth & Semenza 1995). Second, as discussed above, the presence of number attraction on its own cannot provide conclusive insight into the route used to determine pronoun form, as number has conceptual correlates. Grammatical gender attraction would be a better test, as it would show that pronoun attraction errors arise due to the influence of a linguistic element, which can be parsimoniously explained by the syntactic route but not the conceptual-lexical route. It is unclear whether the grammatical gender attraction effects that have so far only been elicited with a preamble task (Meyer & Bock 1999) would persist in a scene description task that is less likely to artificially favor the syntactic route to pronoun form planning.

### The present study

The present study investigates how speakers determine pronoun form, using agreement attraction effects to arbitrate between the conceptual-lexical and the syntactic routes. We elicited pronouns using a scene description task inspired by that of Kandel, Wyatt & Phillips (2024), allowing us to investigate how pronoun form is determined when the challenges for the speaker are more similar to those in natural speech than in prior preamble completion experiments (e.g., Bock, Nicol & Cutting 1999; Meyer & Bock 1999). The study comprises two experiments that tested pronoun agreement attraction in Spanish, a language with rich inflectional morphology whose pronouns bear both number and grammatical gender features.

Experiment 1 tested whether the number attraction findings observed in prior studies replicate in Spanish, allowing us to assess the reliability of the effect cross-linguistically and whether there are cross-linguistic differences in pronoun form planning. As discussed above, findings from subject–verb agreement studies suggest that speakers of languages with richer inflectional morphology than English rely more on conceptual number. If this extends to antecedent–pronoun agreement, Spanish speakers may use the conceptual-lexical route more and potentially avoid attraction. Alternatively, the findings with subject–verb agreement might not extend to pronouns — these findings have been attributed to the availability of tacit subjects in Spanish/Italian, but this may not be relevant for antecedent–pronoun dependencies. If so, other outcomes are conceivable. For instance, Spanish speakers may be more likely than English speakers to use a syntactic route to pronoun form, potentially showing more attraction: This is because Spanish pronouns not only contain features with conceptual correlates (number, natural gender) but must also agree with the grammatical gender of their antecedent (a linguistic feature).

Experiment 2 tested whether Spanish pronouns show grammatical gender attraction, providing additional insight into the source of pronoun attraction effects. If we observe both number and gender attraction effects, that would provide evidence of an influence from linguistic representations, suggesting that pronouns are planned using the syntactic route. By contrast, if we observe number attraction but no grammatical gender attraction, it is possible that pronoun attraction errors only arise due to interference at the conceptual level, since number (but not grammatical gender) has conceptual correlates. By eliciting both forms of attraction using the same paradigm within the same language, we can be confident that any differences we observe between Experiments 1 and 2 are due to the pronoun feature tested (number vs. gender) as opposed to differences between studies or languages.

Following Kandel, Wyatt and Phillips (2024), we diagnosed attraction using two dependent measures. The first was the presence of agreement errors (the typical measure used to assess attraction). The second was the duration of the post-attractor region in error-free sentences. Previous work with the scene description paradigm has shown that the time taken to produce an agreement target can provide insight into how often attraction pressures are active, even in cases when no error is made (Kandel & Phillips 2022; Kandel, Wyatt & Phillips 2022). Consequently, by assessing attraction with two different measures, we may be able to derive greater insight into how often and in what contexts the processes and pressures that underlie pronoun number and gender attraction effects are active.

## Experiment 1: Number Attraction

### Methods

#### Participants

Experiment 1 had a sample of 47 native speakers of Spanish who were born in Spain and located there at the time of testing (23 female, 23 male, 1 other). Participants had a mean age of 29 years (SD = 8.2 years) and reported no language, vision or auditory impairments. They were recruited through the online platform Prolific (www.prolific.com) and received monetary compensation for their participation. An additional nine participants completed the study but were excluded from the analysis due to poor sound quality in their recordings, technical difficulties preventing completion of the experiment, or producing pronouns in less than 30% of their responses.

#### Materials

Participants described videos of inanimate objects touching other objects and turning them black. This action was referred to with the made-up verb *pipear* (‘pipping’). This word was based on the nonce word *mimmed* used by Kandel et al. (2024), except that the initial phoneme was replaced by a plosive in order to facilitate onset detection in the latency analysis. We used a non-word to discourage participants from preceding the direct objects of their sentences with the differential object marking preposition *a* (see Von Heusinger & Kaiser 2007 for the relationship between differential object marking and the lexical semantics of verbs in Spanish). The use of differential object marking may contribute to participants’ perception of objects as animate and, relatedly, female or male (see von Heusinger & Kaiser 2003 for the connection between differential object marking and animacy, and Fábregas 2013 for an overview of differential object marking in Spanish). This was important for Experiment 2, which aimed to test whether grammatical (rather than natural) gender elicits agreement attraction.^1^

The elicited sentences had the target structure: NP1 (antecedent) + verb *pipear* + NP2 (attractor) + *above/below* + pronoun. We manipulated the number of the antecedent noun (singular/plural) and whether the antecedent and attractor nouns matched in number (match/mismatch), resulting in four experimental conditions (2 match conditions and 2 mismatch conditions; Table 1).

**Table 1:** Example target sentences in Experiment 1.


CONDITION	TARGET SENTENCE

*SS – match*	**El chaleco** ha pipeado el candado (de) debajo de **él** *The vest has pipped the lock below it*

*SP – mismatch*	**El chaleco** ha pipeado los candados (de) debajo de **él** *The vest has pipped the locks below it*

*PP – match*	**Los chalecos** han pipeado los candados (de) debajo de **ellos** *The vests have pipped the locks below them*

*PS – mismatch*	**Los chalecos** han pipeado el candado (de) debajo de **ellos** *The vests have pipped the lock below them*


*Note*. The antecedent and coreferential pronoun are bolded, while the attractor is underlined. The preposition “de” before the adverb “debajo” is shown between parentheses to reflect its optional status: In pilot testing, some Spanish speakers prefered to produce it, while others didn’t **(Supplemental file 1)**. Therefore, both utterances with and without the preposition were accepted as target responses in Experiment 1. Abbreviations: SS = singular antecedent, singular attractor, SP = singular antecedent, plural attractor, PP = plural antecedent, plural attractor, PS = plural antecedent, singular attractor.

Sixteen nouns were used as antecedents and attractors (8 masculine and 8 feminine), leading to 112 target sentences (28 per participant per condition; half with masculine nouns and half with feminine nouns; within a sentence, all nouns were the same gender). The nouns were all three syllables with stereotypical gender suffixes (*-o* for masculine and *-a* for feminine). Because the same nouns were used in Experiment 2 (which manipulated gender), care was taken to ensure that masculine and feminine nouns had similar frequency values. Based on the Subtlex-ESP database (Cuetos et al. 2011), masculine nouns had an average raw frequency per million words of 17.47 (range: 3.03–41.54) and feminine nouns of 17.01 (range: 4.52–43.07) (t(14) = –0.07, p = 0.95).^2^

To create videos corresponding to each target sentence, we used images corresponding to each noun from the MultiPic database (Duñabeitia et al. 2018). The images had intermediate visual complexity on a 1–5 scale (average: 1.92, range: 1.4–2.29), and there was no evidence that complexity differed across masculine and feminine nouns (masculine nouns average: 1.89, range: 1.5–2.29; feminine nouns average: 1.98, range: 1.4–2.29; t(14) = 0.62, p = 0.54). The images all had high naming consistency (average: 99%, range: 94–100%), though naming consistency was slightly higher for feminine nouns (masculine nouns average: 98%; range: 94–100%; feminine nouns average: 100%, range: 100–100%; t(14) = 2.17, p = 0.048). This difference is unlikely to have affected the study results, as participants were introduced to the target names for all images at the start of the experiment (see *Procedure* below).

**Figure 2:**
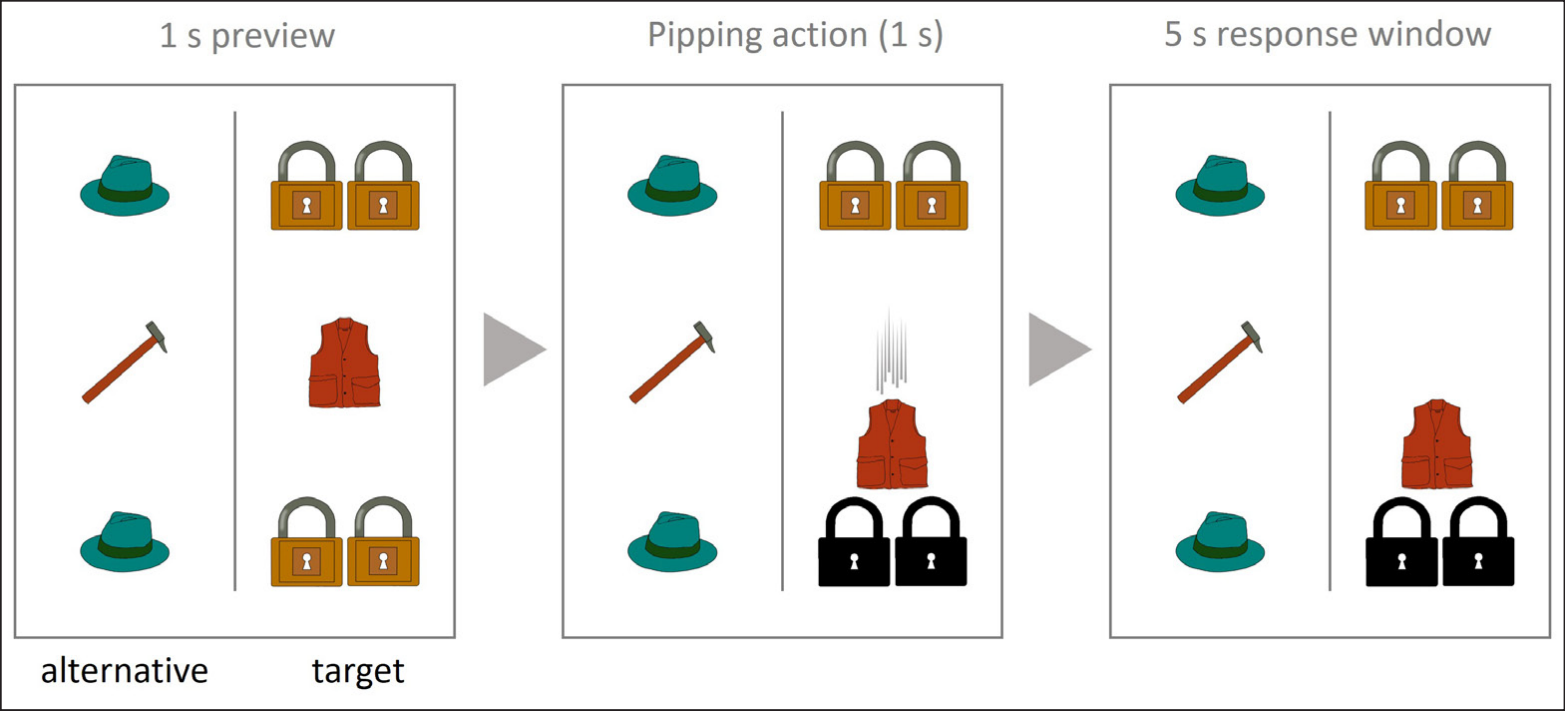
Example of a visual display in Experiment 1. *Note*. The display corresponds to the target sentence “El chaleco ha pipeado los candados debajo de él” (‘The vest has pipped the locks below it’). A trial consisted of a 1 second preview, the pipping action, and then a 5 second response window in which participants had to describe the action.

Each video was divided into a target and an alternative display. This was done to prevent participants from planning their responses before the pipping action occurred. In each display, a central object set (consisting of one or two objects of the same type) was positioned with two identical sets of objects above and below it (Figure 2). In the target display, this central object set (corresponding to N1; the antecedent) pipped the object set above or below it (corresponding to N2; the attractor). The position of the target display (left/right) was counterbalanced across items. If the target display corresponded to a match condition, the alternative corresponded to a mismatch condition, and vice versa. This was done to avoid some trials being perceived as more difficult than others. We made sure that the position of the attractor in the target display (above or below the antecedent) was evenly distributed across trials. The nouns representing the on-screen objects always had the same gender (across both the target and alternative displays), to avoid mixing number and gender interference.

#### Procedure

The experiment was conducted using PCIbex (Zehr & Schwarz 2018). Responses were recorded using the participants’ microphones. Recording started and ended automatically for each trial. Participants were told that they would see scenes from a spinoff game of Tetris called *The Haunted Attic*, where there were sixteen different objects (see the OSF repository for a transcript of the instructions). They were introduced to the objects and their target names, as well as to the pipping action that the objects performed when they interacted with each other, which was both described and shown in video examples. Participants were then told that their task was to describe ‘what pipped what’, and that there would be several objects in the scenes, meaning that they would need to use *encima de* (‘above’) and *debajo de* (‘below’) to make their descriptions more precise. Participants were not asked to integrate these adverbial phrases in their utterances in a specific way, and they were also not asked to use pronouns, but they were given examples that followed the structure of the sentences in Table 1. Subsequently, they completed seven practice trials. Each trial started with a one second preview, followed by the pipping action, which was accompanied by a sound. Afterwards, participants articulated their responses. The first two practice trials were not timed, and participants pressed a button after giving their response to end the trial; at the end of each trial, participants were presented with the suggested target sentence. The final five practice trials followed the same procedure as the experimental items: Participants had five seconds to articulate their response before the trial (and recording) ended automatically, and no suggested target sentences were presented. The experimental trials were pseudorandomized for each participant to prevent showing two consecutive trials with the same condition or pronoun. An experimental session lasted on average 30 minutes.

### Analysis

#### Error analysis

The error analysis assessed whether the likelihood of pronoun number errors differed across conditions. Responses were manually transcribed and coded for whether they matched the target response and whether they contained a pronoun number error. Pronoun number errors included both unrevised and revised errors. Responses were considered non-target and excluded from analysis if they were incomplete, if the pronoun was unintelligible, or if they contained incorrect noun or verb number, lexical substitutions (*shield* instead of *hat*, or *above* instead of *below*), a pronoun gender error, or late corrections at the end of the utterance (e.g., “*The hat pipped the sword below him… the shield*”), with the exception of pronoun revisions. Responses were considered target if the target nouns were replaced by other nouns with similar meaning and the same gender and number of syllables (e.g., *los cerrojos* for *los candados*), if they contained a disfluency (e.g., false starts, word repetitions, etc.), or if the differential object marking preposition *a* preceded the attractor (e.g., *a los chalecos* instead of *los chalecos*).

All statistical analyses in the present study were performed using the package *lme4* (v.1.1.35.1; Bates et al. 2015) in R (version 4.3.3, R Core Team 2024). We examined the probability of pronoun number errors with binomial (logistic) mixed effects regression. Errors were coded as 1, and accurate responses were coded as 0. The predictors were Antecedent Number (sum-coded, –0.5 singular/0.5 plural), Match (sum-coded, –0.5 match/0.5 mismatch), and their interaction, as well as a centered numeric predictor: Trial Order. The random effects structure included intercepts by subject and item and slopes for Antecedent Number, Match, and their interaction, which were the predictors of theoretical interest. The simultaneous inclusion of by-participant and by-item random effects allowed us to account for potential differences in the properties of the images and/or nouns across the experimental items. If necessary, the random effect structure was simplified to achieve convergence. We report the final structures in the tables showing the models’ output.

#### Latency analysis

**Figure 3:**
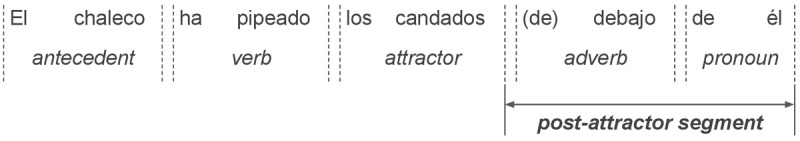
Example of a target response with its division into segments.

The latency analysis assessed whether there were articulation slowdowns in utterances with correct pronoun forms across conditions. The latency analysis excluded all non-target utterances as well as utterances with disfluencies and number errors — i.e., only complete and correct responses were included in the analysis. These responses were aligned to their transcriptions at the word boundary level using the Montreal Forced Aligner (v.2.0.0; McAuliffe et al. 2017). We used the word onsets and offsets identified by the forced aligner to compute the duration of the post-attractor segment.^3^ The post-attractor segment duration was computed by subtracting the offset of the pronoun from the offset of the attractor noun (Figure 3). This segment was chosen because the attractor offset was the earliest point in time in which the pronoun form could be planned without interference from the planning of the two preceding noun phrases. The performance of the forced-aligner was evaluated by comparing its alignments to those of two humans and computing inter-rater agreements for word boundaries **(Supplemental file 2)**. The interquartile range criterion was used to detect outliers (in ms), such that observations below the first quartile or above the third quartile by 1.5 times the interquartile range were excluded (Hawkins 1980).

The statistical analysis examined post-attractor segment latencies with linear mixed effects regression, using the same predictors as in the error analysis, with the addition of Syllable Count (the number of syllables of the post-attractor segment). Syllable Count was included in the model to account for the fact that plural pronouns were one syllable longer than singular pronouns. The dependent variable was the log-transformed duration of the post-attractor segment.

In both the error and latency analyses, if there was a significant interaction, the model was re-fit such that the interaction term was replaced by the nested effects of the critical factor (e.g., the effect of Match for singular and plural antecedents separately).

### Results

Out of 5,264 utterances, 8.03% were incomplete (range across conditions: 4.94–10.3%). Of the remaining 4,841 complete utterances, 5.41% were excluded from the error analysis due to containing non-target responses (range across conditions: 4.41–6.02%), and 12.95% were excluded from the latency analysis due to containing non-target responses and/or number errors (range across conditions: 10.2–15.3%). No observations were excluded from the latency analysis as outliers after the application of the interquartile range criterion. Thus, 4,579 trials were entered into the error analysis, and 4,214 trials were entered into the duration analysis.

#### Error analysis

**Figure 4:**
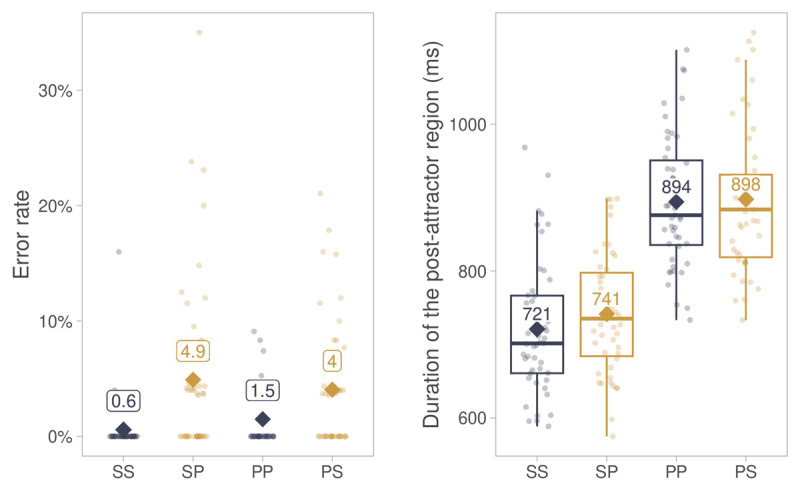
Descriptive summary of error rates and durations of the post-attractor segment in Experiment 1. *Note*. Diamonds show averages across participants in match (SS, PP) and mismatch (SP, PS) conditions. Points show by-participant averages. Abbreviations: SS = singular antecedent, singular attractor, SP = singular antecedent, plural attractor, PP = plural antecedent, plural attractor, PS = plural antecedent, singular attractor.

The distribution of number error rates across conditions is shown in Figure 4. The statistical analysis showed an effect of match (Table 2): Number errors were more likely in mismatch than in match conditions, consistent with agreement attraction. Further, there was an interaction between antecedent number and match. Nested comparisons showed that the effect of match was larger with singular antecedents (estimate = 2.230, *z* = 5.384, *p* < 0.001) than with plural antecedents (estimate = 1.074, *z* = 3.585, *p* < 0.001). This suggests that plural attractors (as in the SP condition) caused more attraction than singular attractors (as in the PS condition), consistent with a number markedness asymmetry.

**Table 2:** Output of the Experiment 1 error analysis model.


COEFFICIENT	ESTIMATE	STANDARD ERROR	z-value	p-value

Intercept (grand mean)	–4.430	0.234	–18.939	< 0.001

Trial Order	–0.004	0.003	–1.368	0.171

Antecedent Number	0.487	0.309	1.577	0.115

Match	1.651	0.260	6.350	< 0.001

Antecedent Number × Match	–1.158	0.512	–2.259	0.024


*Note*. Model formula: Number Error ~ Trial Order + Antecedent Number * Match + (1 + Antecedent Number * Match || Participant) + (1 + Match || Item). A positive coefficient for Antecedent Number reflects more number errors with plural than singular antecedents. A positive coefficient for Match reflects a greater likelihood of number errors for mismatch than match conditions. The double bars in the model formula represent the removal of the correlation between random slopes and intercepts.

#### Latency analysis

The distribution of durations in the post-attractor segment is shown in Figure 4. The statistical analysis showed a main effect of Match (Table 3): The duration of the post-attractor segment was longer in the mismatch than in the match conditions, consistent with agreement attraction. There was also a main effect of antecedent number, with longer durations for trials with plural vs. singular antecedents. Finally, durations increased with increasing number of syllables.

**Table 3:** Output of the Experiment 1 latency analysis model.


COEFFICIENT	ESTIMATE	STANDARD ERROR	t-value	p-value

Intercept (grand mean)	6.684	0.013	527.406	< 0.001

Trial Order	–0.000	0.000	–2.654	0.008

Syllable Count	0.096	0.004	25.513	< 0.001

Antecedent Number	0.158	0.006	25.98	< 0.001

Match	0.015	0.004	3.941	< 0.001

Antecedent Number × Match	–0.020	0.013	–1.489	0.143


*Note*. Model formula: log(Duration) ~ Trial Order + Syllable Count + Antecedent Number * Match + (1 + Antecedent Number * Match | Participant) + (1 + Antecedent Number * Match | Item). A positive coefficient for Antecedent Number reflects longer durations for post-attractor segments with plural than singular antecedents. A positive coefficient for Match reflects longer durations for mismatch than match conditions.

### Discussion

The results of Experiment 1 show that the number agreement attraction effect observed in prior experiments in English replicates in Spanish. We observed evidence for agreement attraction in both error and timing measures: Number agreement errors were more likely in the mismatch conditions, and speakers were slower to articulate the post-attractor region containing the pronoun in these conditions even when the correct pronoun was produced, suggesting that the process leading to errors is active on more than just the trials in which errors occur (see Kandel, Wyatt & Phillips 2022 for discussion of how to interpret attraction timing effects for verbs). The error effect showed a similar markedness asymmetry to that observed in verb attraction studies, with stronger attraction from plural attractors than singular ones (e.g., Bock et al. 2006; Bock & Miller 1991; Bock, Nicol & Cutting 1999; Eberhard 1997; Thornton & MacDonald 2003; Vigliocco & Nicol 1998).

The results illustrate that representations other than the pronoun antecedent can interfere when determining pronoun number. As discussed in the Introduction, this influence is suggestive of the use of a syntactic route to determine pronoun features. However, since number has conceptual correlates, we cannot conclusively infer that it was the representation of the attractor at the sentence-level rather than the message-level that caused attraction. Grammatical gender, which doesn’t have systematic conceptual correlates, thus serves as a stronger test of whether attraction can arise from linguistic representations other than that of the antecedent. Experiment 2 assessed the presence of grammatical gender attraction.

## Experiment 2: Gender Attraction

### Methods

#### Participants

Experiment 2 had a sample of 72 native speakers of Spanish who were born in Spain and located there at the time of testing (33 female, 38 male, 1 other). Participants had a mean age of 29.2 years (SD = 7.1 years) and reported no language, vision or auditory impairments. They were recruited through the online platform Prolific (www.prolific.com) and received monetary compensation for their participation. An additional five participants were excluded from the analysis due to poor sound quality in their recordings, technical difficulties preventing completion of the experiment or saving of the recordings, or producing pronouns in less than 30% of their responses.

Note that Experiment 2 had a larger sample size than Experiment 1 (N = 47). Given that pronoun gender attraction effects in prior literature (Meyer & Bock 1999) seem descriptively smaller than number attraction effects elicited with a similar paradigm (Bock, Nicol & Cutting 1999; Bock, Eberhard & Cutting 2004; Bock et al., 2006), we collected a larger sample in order to increase our likelihood of detecting a gender attraction effect.

#### Materials and procedure

Experiment 2 closely followed the format of Experiment 1, with a few modifications to fit a gender attraction paradigm. The elicited target sentences used the same structure and nouns as Experiment 1. Our design had four experimental conditions as a result of manipulating the gender of the antecedent and the attractor (Table 4). However, unlike Experiment 1, an item was defined as a set of pairings with the same antecedent. Consequently, the materials consisted of 16 items. For each item, we combined the antecedent with seven matching and seven mismatching nouns, creating 224 combinations. We excluded one possible mismatch combination from all items to ensure that all nouns would be equally distributed across antecedent and attractor positions. These combinations were used for practice trials and examples. 224 videos were created using the same images and following the same format as in Experiment 1. These were distributed into two presentation lists such that each participant saw 112 videos, as in Experiment 1. Participants were evenly distributed across these two presentation lists, meaning that we elicited 36 responses to each target sentence. The two lists balanced the number of match and mismatch conditions, the position of the pipped object (below/above), the distribution of masculine and feminine nouns in the corresponding target sentences, and how many times each noun appeared in antecedent and attractor positions. The procedure was the same as in Experiment 1, except that the pipping sound was removed to avoid potential interference in the forced-alignment process.

**Table 4:** Example target sentences in Experiment 2.


CONDITION	TARGET SENTENCE

*MM – match*	**El chaleco** ha pipeado el candado (de) debajo de **él** *The vest_MASCULINE_ has pipped the lock_MASCULINE_ below it*

*MF – mismatch*	**El chaleco** ha pipeado la medalla (de) debajo de **él** *The vest_MASCULINE_ has pipped the medal_FEMININE_ below it*

*FF – match*	**La medalla** ha pipeado la lámpara (de) debajo de **ella** *The medal_FEMININE_ has pipped the lamp_FEMININE_ below it*

*FM – mismatch*	**La medalla** ha pipeado el candado (de) debajo de **ella** *The medal_FEMININE_ has pipped the lock_MASCULINE_ below it*


*Note*. The antecedent and coreferential pronoun are bolded, while the attractor is underlined. Abbreviations: MM = masculine antecedent, masculine attractor, MF = masculine antecedent, feminine attractor, FF = feminine antecedent, feminine attractor, FM = feminine antecedent, masculine attractor.

### Analysis

Responses were transcribed and coded in the same way as in Experiment 1, except that we coded gender errors instead of number errors (note that participants did not produce any pronoun number errors). The statistical analysis also followed Experiment 1, except that the predictor Antecedent Number was replaced with Antecedent Gender (sum-coded, –0.5 masculine/0.5 feminine). Since the gender of the antecedent noun did not vary within items, no by-item random slope for Antecedent Gender was entered in the random structure of the statistical models.

### Results

Out of 8,064 utterances, 4.3% were incomplete (range across conditions: 3.72–4.81%). Of the remaining 7,720 complete utterances, 1.68% were excluded from the error analysis due to containing non-target responses (range across conditions: 1.08–2.08%), and 12.23% were excluded from the latency analysis due to containing non-target responses and/or gender errors (range across conditions: 10.6–15.6%). The removal of outliers for the latency analysis using the interquartile range criterion excluded 2.64% of the remaining data (range across conditions: 2.48–2.99%). Thus, 7,590 trials were entered into the statistical analysis of gender errors, and 6,597 trials were entered into the analysis of post-attractor segment durations.

#### Error analysis

**Figure 5:**
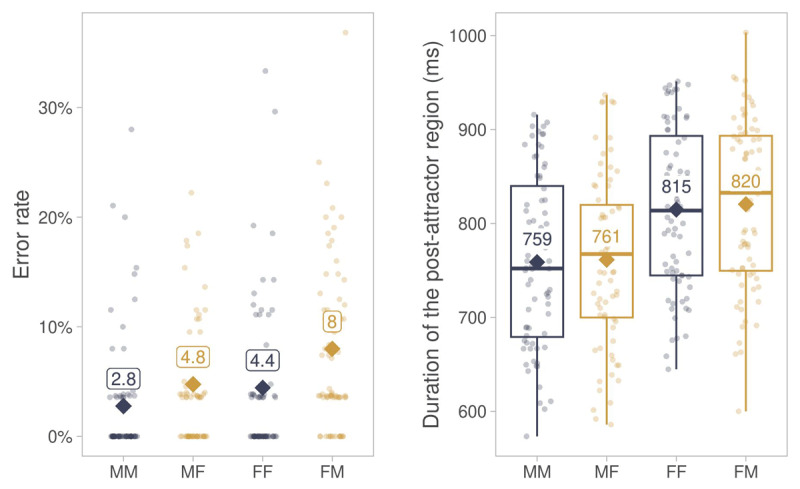
Descriptive summary of error rates and durations of the post-attractor segment in Experiment 2. *Note*. Diamonds show averages across participants in match (MM, FF) and mismatch (MF, FM) conditions. Points show by-participant averages. Abbreviations: MM = masculine antecedent, masculine attractor, MF = masculine antecedent, feminine attractor, FF = feminine antecedent, feminine attractor, FM = feminine antecedent, masculine attractor.

The distribution of gender error rates across conditions is shown in Figure 5. The statistical analysis showed positive main effects of Match and Antecedent Gender (Table 5). Errors were more likely in mismatch than in match conditions, consistent with agreement attraction, and with feminine than masculine antecedents.

**Table 5:** Output of the Experiment 2 error analysis model.


COEFFICIENT	ESTIMATE	STANDARD ERROR	z-value	p-value

Intercept (grand mean)	–3.437	0.149	–23.003	< 0.001

Trial Order	0.001	0.002	0.768	0.443

Antecedent Gender	0.567	0.236	2.406	0.016

Match	0.730	0.144	5.068	< 0.001

Antecedent Gender × Match	0.051	0.229	0.222	0.824


*Note*. Model formula: Gender Error ~ Trial Order + Antecedent Gender * Match + (1 + Antecedent Gender * Match || Participant) + (1 + Match || Item). A positive coefficient for Antecedent Gender reflects a greater likelihood of gender errors with feminine than masculine antecedents. A positive coefficient for Match reflects a greater likelihood of gender errors for mismatch than match conditions. The double bars in the model formula represent the removal of the correlation between random slopes and intercepts.

#### Latency analysis

The distribution of durations in the post-attractor segment is shown in Figure 5. The statistical analysis showed no evidence of attraction, as the effect of Match did not reach significance (Table 6). There was an effect of Antecedent Gender, with shorter post-attractor durations in trials with feminine than masculine antecedents. Note that this is the opposite direction than suggested by Figure 5 — when Syllable Count was added to the model, the effect reversed in direction. Finally, durations decreased with increasing Trial Order and increased with increasing Syllable Count.

**Table 6:** Output of the Experiment 2 error analysis model.


COEFFICIENT	ESTIMATE	STANDARD ERROR	t-value	p-value

Intercept (grand mean)	6.658	0.010	670.802	< 0.001

Order	–0.000	0.000	–4.275	< 0.001

Syllable Count	0.132	0.006	22.250	< 0.001

Antecedent Gender	–0.055	0.009	–6.449	< 0.001

Match	0.002	0.004	0.676	0.501

Antecedent Gender × Match	0.003	0.006	0.446	0.657


*Note*. Model formula: log(Duration) ~ Trial Order + Syllable Count + Antecedent Gender * Match + (1 + Antecedent Gender * Match || Participant) + (1 + Match || Item). A negative coefficient for Antecedent Gender reflects shorter durations of the post-attractor segment in feminine than masculine conditions. A positive coefficient for Match reflects longer durations in mismatch than match conditions. The double bars in the model formula represent the removal of the correlation between random slopes and intercepts.

#### Exploratory analysis: Comparison of number and gender attraction

In order to directly compare gender and number attraction effects, two additional models were run combining the error and latency data from the two experiments (see **Supplemental file 3** for full model outputs). For the error analysis, a mixed logistic regression model was run using as predictors Trial Order (centered), Match (–0.5 mismatch/+0.5 match), Experiment (–0.5 experiment 1/+0.5 experiment 2), and the interaction between Match and Experiment. This Match × Experiment interaction was the critical predictor, because it directly quantified the difference in the size of attraction effects for number vs. gender. By-participant and by-item random intercepts and slopes for Match were included in the model. The results of the error analysis showed an interaction between Experiment and Match (estimate = –0.793, *z* = –2.643, *p* = 0.008). Nested comparisons revealed a greater effect of match — i.e., a larger attraction effect — in the number experiment (estimate = 1.812, *z* = 5.95, *p* < 0.001) than in the gender experiment (estimate = 0.932, *z* = 5.262, *p* < 0.001).

For the latency analysis, a mixed linear regression model was run on the combined latency data using as predictors Trial Order (centered), Syllable Count (centered), Match (–0.5 mismatch/+0.5 match), Experiment (–0.5 experiment 1/+0.5 experiment 2), and the interaction between Match and Experiment. By-participant and by-item random intercepts and slopes for Match were included in the model. The results showed an interaction between Experiment and Match (estimate = –0.014, *t* = –2.223, *p* = 0.028). Nested comparisons showed that the effect of match was only significant in Experiment 1 (the number experiment) (estimate = 0.016, *t* = 3.224, *p* = 0.002), not in Experiment 2 (the gender experiment) (estimate = 0.002, *t* = 0.457, *p* = 0.648). Thus, both the error and duration analyses showed evidence of stronger attraction effects with number than with gender.

### Discussion

Experiment 2 showed evidence of grammatical gender attraction in the error analysis: Gender agreement errors were more likely in the mismatch than match conditions. Since grammatical gender doesn’t have conceptual correlates, grammatical gender attraction is indicative of influence from the linguistic representation of the attractor, suggesting that speakers use a syntactic route to determine pronoun form (at least at times).

To our knowledge, Experiment 2 represents the first time that a gender attraction effect in pronouns has been elicited using a scene description paradigm, as opposed to a preamble paradigm (e.g., Meyer & Bock 1999). Although the overall rate of pronoun gender errors was lower in Experiment 2 (3–8%; see **Supplemental file 3**) than in Meyer and Bock (1999, Experiment 1) (9–30%), the size of the attraction effect was not smaller: Compared to the match conditions, the mismatch conditions elicited 1.8–1.9x more errors in our Experiment 2 and 1.2–1.5x more errors in Meyer and Bock’s (1999) most analogous sentence construction (Experiment 1, with definite determiners and the antecedent as the first of the two noun phrases in the sentence).

The gender attraction errors in our Experiment 2 were also not accompanied by a latency effect, in contrast to the number attraction errors in Experiment 1. This might indicate that the pressures leading to gender errors are weaker and/or not as active as the pressures leading to number errors. Gender attraction also differed from number attraction in that it showed no markedness asymmetry, i.e., attraction rates did not differ across genders. We discuss potential sources of these differences further in the General Discussion.

## General Discussion

Our study used agreement attraction to investigate how speakers determine pronoun form. We considered two possible routes to pronoun form: a syntactic route, whereby pronouns obtain their features from the linguistic antecedent, and a conceptual-lexical route, whereby pronouns get their features from the concept of the referent — either directly from the concept itself (direct path) or via an associated lemma in the mental lexicon (mediated path). As discussed in the Introduction, the conceptual-lexical route should be robust to errors (independent of the path used), which would not predict the occurrence of attraction effects. By contrast, the use of a syntactic route more readily predicts agreement attraction.

We conducted our study in Spanish, a language with productive inflectional morphology whose pronouns agree with their antecedents in both number and grammatical gender, thereby allowing us to test the presence of number (Experiment 1) and grammatical gender attraction (Experiment 2). We elicited sentences using a scene description paradigm similar to one previously applied in English to study number attraction (Kandel & Phillips 2022; Kandel, Wyatt & Phillips 2024). An advantage of this paradigm is that it is less likely to bias the use of the syntactic route than the preamble elicitation paradigm traditionally used in prior work. The same paradigm was used in both experiments, allowing us to compare number and gender attraction rates.

We observed attraction with both number and grammatical gender features: Pronoun agreement errors were more likely when an attractor differed from the antecedent in number or gender. For number, we additionally observed slowing during the articulation of the part of the sentence containing the pronoun (the post-attractor segment) in the mismatch conditions when the correct form of the pronoun was produced, which could reflect slowing due to internal revision and/or increased planning difficulty (Kandel, Wyatt & Phillips 2022). These findings can be explained under a syntactic route, as attraction indicates that pronoun form can be influenced by something other than the concept of its referent. Searching for an antecedent involves accessing a representation at the sentence level, which also contains the attractor, thereby enabling it to influence pronoun form. The use of a syntactic route is particularly supported by the presence of gender attraction, as grammatical gender does not have conceptual correlates and therefore attraction must have arisen due to influence from a linguistic representation.

Our results extend previous English findings (Kandel & Phillips 2022; Kandel, Wyatt & Phillips 2024) and demonstrate that pronoun attraction also occurs in a language with productive inflectional morphology. Spanish participants might have been expected to be resilient to attraction, given that speakers of morphologically rich languages have been reported to rely more on conceptual number, consistent with an increased reliance on a conceptual-lexical route (Vigliocco 1996; Vigliocco, Butterworth & Garrett 1996; Vigliocco, Butterworth & Semenza 1995). However, our results suggest that this was not the case. If attraction error rates are taken as support for the involvement of a syntactic route, then the presence of attraction in both English and Spanish suggests that the syntactic route is applied cross-linguistically.

### Differences between number and grammatical gender attraction

There were differences between the observed number and gender attraction effects, both in size and shape. Specifically, attraction was larger for number than grammatical gender, both in error and latency measures. These results suggest that the pressures leading to number attraction are more prevalent and/or stronger than those leading to gender attraction (Eberhard, Cutting & Bock 2005). This increased pressure could in part result from the fact that number has conceptual correlates, meaning that the attractor number may be able to influence agreement at both the message level and the linguistic level (see next section for discussion). The asymmetry could also be related to the fact that, in our experiments, number is extrinsic while grammatical gender is intrinsic, i.e., non-inflectional and deriving from the word itself (Eberhard, Cutting & Bock 2005). It is possible that the process of inflecting a word boosts its representation in working memory (Bock et al., 2001), resulting in more interference from attractors with extrinsic features (e.g., Bock, Eberhard & Cutting 2004) — in this case, the larger number attraction effect would be primarily driven by an increase in errors when the attractor is plural (note that we observed greater attraction from plural attractors than singular ones, more below).

Further, number and gender attraction effects displayed a different pattern. In Experiment 1, we observed a markedness asymmetry for number, similar to that previously observed for verb attraction (Bock et al. 2006; Bock & Miller 1991; Bock, Nicol & Cutting 1999; Eberhard 1997; Thornton & MacDonald 2003; Vigliocco & Nicol 1998): Attraction effects were larger with plural than singular attractors, as indicated by the interaction between match and antecedent number in the error analysis. We did not observe an analogous markedness effect with grammatical gender: The interaction between match and antecedent gender was not reliable. Previous investigations of grammatical gender asymmetries for verb agreement are mixed, with some studies finding asymmetries (Antón-Méndez, Nicol & Garrett 2002; Badecker & Kuminiak 2007; Franck et al. 2008; Slioussar & Malko 2016; Vigliocco & Franck 1999, Experiment 1) but others not (Igoa, García-Albea & Sánchez-Casas 1999; Vigliocco & Franck 1999, Experiment 2). Eberhard, Cutting & Bock (2005) suggest that a reduced gender-based asymmetry could arise due to differences in contrastiveness for gender and number — similar to the argument used by Bock et al. (2001) to explain differences between intrinsic and extrinsic attraction.

Alternatively, a gender markedness asymmetry may have been obscured by a tendency for participants in Experiment 2 to generalize the use of the masculine pronoun, as indicated by the main effect of antecedent gender (more errors with feminine antecedents). If speakers were more likely to produce masculine than feminine pronouns, this would lead to more errors in sentences with feminine antecedents and masculine attractors and fewer errors in sentences with masculine antecedents and feminine attractors. Because this asymmetry works in the opposite direction from what would be expected of a gender markedness asymmetry (more errors in sentences with masculine antecedents and feminine attractors), it may have canceled it out. Such generalization of the masculine pronoun could arise because masculine is considered the default gender in Spanish (Prado 1982; see Beatty-Martínez & Dussias 2019 for review). If grammatical gender is encoded as a feature node associated with lemmas (Levelt, Roelofs & Meyer 1999) and masculine is the default, the masculine feature may have higher resting activation than the feminine feature in working memory, thereby leading to a higher overall likelihood of producing masculine pronouns. Note, however, that by the same logic, we might have expected participants to generalize the use of the singular pronoun, to the extent that singular is the default unmarked number in Spanish. This was not the case in the present study, though this pattern was observed by Kandel et al. (2024).

### Determining pronoun form

Our results provide insight into how speakers determine pronoun form. In particular, participants in our study appeared to use a syntactic route to pronoun form, identifying the required features through a feature-matching process with a linguistic antecedent. Accessing the antecedent for feature-matching opens up the opportunity for other sentence material to interfere, which can explain the observed attraction effects. Nevertheless, participants still used the correct pronoun form on most trials. These low error rates could occur if the syntactic route is very robust to interference or if speakers use a mix of routes to determine pronoun form (Kandel, Wyatt & Phillips 2024). As mentioned in the Introduction, speakers may need different routes for different contexts in which they produce a pronoun (e.g., pronominalization when there is no linguistic antecedent vs. pronominalization with an intra-sentential antecedent), so both routes may be available for speakers. If participants in our experiments used the conceptual-lexical route on a subset of trials, then they would make fewer errors than if they routinely used the syntactic route.

Although a syntactic route to pronoun form has been considered in prior literature (e.g., Meyer & Bock 1999), the real-time mechanisms used to implement the pronoun–antecedent feature matching process within this route remain unclear. Eberhard, Cutting & Bock (2005) propose that pronouns derive their features in part through a marking-and-morphing operation with the antecedent, similar to that used for subject–verb agreement. In particular, they suggest that during structural integration pronouns reference a representation of the antecedent’s number value that is continuous from singular to plural (rather than having a binary singular or plural distinction), with the plurality of this value depending on the notional and morphological number of the noun(s) within the antecedent phrase. Agreement attraction arises when this value is more ambiguous (e.g., when an antecedent phrase contains multiple nouns of different number), leading agreement errors to be probabilistically produced (Eberhard, Cutting & Bock 2005). However, marking-and-morphing does not readily account for our results because the antecedents in our experiments contained a single noun (i.e., the attractor noun was not part of the subject phrase), and thus their number should have been unambiguous to speakers.^4^

Kandel, Wyatt & Phillips (2024) propose an alternative model of pronoun formulation, in which speakers engage a retrieval process for feature matching. Speakers retrieve the linguistic representation of the antecedent from memory and then use its features to determine pronoun form (for similar retrieval accounts of subject–verb agreement in production see Badecker & Kuminiak 2007; Lorimor, Jackson & Foote 2015; inter alia). Kandel, Wyatt and Phillips (2024) adopt the pronominalization mechanism from Schmitt, Meyer & Levelt (1999), in which an ‘in focus’ feature of the message-level representation of the referent (indicating prominence/salience in the discourse) cues the speaker to produce a pronoun. They suggest that the retrieval process targets representations that have a similar prominent mental status in working memory (e.g., high activation or an ‘in focus’ feature at the lexical level). Within this model, attraction errors arise when the attractor noun’s representation matches (or partially matches) the retrieval cues used to access the antecedent (e.g., if it is similarly highly prominent due to recency of mention), leading it to be erroneously retrieved instead of the antecedent (Kandel, Wyatt & Phillips 2024). This account can explain both the number and grammatical gender attraction effects in the present study, as the attractor noun in the elicited sentences may have had high prominence due to recency of mention and/or the fact that it was being individuated by the prepositional phrase containing the pronoun.

Kandel, Wyatt & Phillips’ (2024) account can also explain the markedness asymmetry observed with number. If inflecting a noun to make it plural (as is necessary for extrinsic number) increases its activation in working memory (Bock et al. 2001), this boost would lead to increased attraction from plural nouns. By contrast, the grammatical gender of the nouns in our study was intrinsic, and thus no comparable inflectional adjustment was necessary, making a gender-based asymmetry less likely. Interestingly, an attraction boost for extrinsic plurals predicts that gender attraction should be greater from plural attractors than from singular ones, assuming that both number and gender agreement rely on the same retrieval process — this prediction can be assessed in future work (Experiment 2 only included singular nouns).

To explain the larger attraction effect for number than gender across experiments, a retrieval account could be integrated with the ideas proposed in the previous section. Specifically, extrinsic features may lead to greater competition from the attractor than intrinsic features (Bock et al. 2001; Eberhard, Cutting & Bock 2005) or conceptual number may also be able to influence agreement. An influence of conceptual number can be incorporated into a retrieval account by combining it with a lexical competition framework, similar to that proposed by Nozari and Omaki (2022) for subject–verb agreement. Within this framework, agreement errors arise due to competition between forms during lexical selection. If candidate pronoun forms are activated not only by the features of the retrieved antecedent (consistent with Kandel, Wyatt & Phillips 2024) but also by the features of nearby nouns (consistent with Nozari & Omaki 2022), then attraction errors can arise either in the case of a retrieval error or if an incorrect pronoun form receives enough activation from a nearby attractor noun to successfully out-compete the correct form. If the message-level number features of nearby nouns contribute to the activation of candidate forms, this will increase activation of competing incorrect forms for number but not gender, thereby leading to a greater number attraction effect.

## Conclusion

We used agreement attraction to contrast two possible routes to pronoun form. Our study elicited pronouns using a scene description paradigm that engages processes involved in natural speech, and it goes beyond prior work with this paradigm by investigating both number and grammatical gender attraction. In particular, since number features have conceptual correlates, testing for grammatical gender attraction allowed us to assess more directly whether pronoun formulation can be disrupted by linguistic representations of nearby nouns. We found small but reliable attraction effects for both features, providing evidence that speakers do not always derive pronoun features from the message-level representation of the referent (the conceptual-lexical route) but rather perform a feature-matching operation that targets the pronoun’s linguistic antecedent (the syntactic route). Nevertheless, speakers likely have both routes available to them to determine pronoun form, which could explain the low incidence of observed errors.

## Data Accessibility Statement

The data and analysis scripts that support the findings of this study are available at https://osf.io/apk4w/.

## Additional Files

The additional files for this article can be found as follows:

10.5334/joc.416.s1 Supplemental File 1. Forced-choice study to select the target structures.

10.5334/joc.416.s2 Supplemental File 2. Evaluation of the forced aligner.

10.5334/joc.416.s3 Supplemental File 3. Supplementary analyses.

## Footnotes

After completion of the study, we learned that the verb *pipear* in fact exists in some varieties of Spanish. However, the verb’s usage and meaning varies, and it is infrequent: there are only four instances — and a single one from Spain — across the Genre/Historical, Web/Dialects, and NOW (2012–2019) sections of the Corpus del Español, which together contain 9.4 billion words (Davies, 2002–, 2016–, 2018–). At the time of writing, *pipear* is not widely-recognized as a Spanish verb — in fact, none of the three authors who are native Spanish speakers were familiar with it. Nevertheless, even if the phonological verb form *pipear* already existed in our participants’ lexicons, we do not anticipate that learning of a novel homophone would have meaningfully affected the likelihood of producing agreement attraction errors.

When using Espal frequency measures (Duchon et al. 2013), the frequency of masculine nouns (average: 15.54, range: 1.07–43.77) and feminine nouns (average: 19.90, range: 2.22–53.89) were also similar (t(14) = 0.50, p = 0.62).

In contrast to Kandel & Phillips (2022), we did not analyze the likelihood of pauses before the agreement target (i.e., the pronoun) because pauses before the pronoun were very infrequent in our data (mean pause likelihood = 0.19%, range across conditions: 0.1–0.4%).

Under Eberhard et al.’s (2005) account, pronoun number errors can also arise when the referent’s notional number is ambiguous. However, this was not the case for the referents in the present study.

## References

[1] Antón-Méndez, I., Nicol, J. L., & Garrett, M. F. (2002). The relation between gender and number agreement processing. Syntax, 5(1), 1–25. 10.1111/1467-9612.00045

[2] Arnold, J. E., & Zerkle, S. A. (2019). Why do people produce pronouns? Pragmatic selection vs. rational models. Language, Cognition and Neuroscience, 34(9), 1152–1175. 10.1080/23273798.2019.1636103

[3] Badecker, W., & Kuminiak, F. (2007). Morphology, agreement, and working memory retrieval in sentence production: Evidence from gender and case in Slovak. Journal of Memory and Language, 56(1), 65–85. 10.1016/j.jml.2006.08.004

[4] Bates, D., Mächler, M., Bolker, B., & Walker, S. (2015). Fitting linear mixed-effects models using lme4. Journal of Statistical Software, 67(1), 1–48. 10.18637/jss.v067.i01

[5] Beatty-Martínez, A. L., & Dussias, P. E. (2019). Revisiting masculine and feminine grammatical gender in Spanish: Linguistic, psycholinguistic, and neurolinguistic evidence. Frontiers in Psychology, 10, 751. 10.3389/fpsyg.2019.00751 31024394 PMC6460095

[6] Bock, K., Cutler, A., Eberhard, K. M., Buttefield, S., Cutting, J. C., & Humphreys, K. R. (2006). Number agreement in British and American English: Disagreeing to agree collectively. Language, 82(1), 64–113. 10.1353/lan.2006.0011

[7] Bock, K., Eberhard, K., Cutting, J. C., Meyer, A. S., & Schriefers, H. (2001). Some attractions of verb agreement. Cognitive Psychology, 43(2), 83–128. 10.1006/cogp.2001.0753 11527432

[8] Bock, K., Eberhard, K. M., & Cutting, J. C. (2004). Producing number agreement: How pronouns equal verbs. Journal of Memory and Language, 51(2), 251–278. 10.1016/j.jml.2004.04.005

[9] Bock, K., & Levelt, W. J. M. (1994). Language production: Grammatical encoding. In M. A. Gernsbacher (Ed.), Handbook of psycholinguistics (pp. 945–984). Academic Press.

[10] Bock, K., & Miller, C. (1991). Broken agreement. Cognitive Psychology, 23(1), 45–93. 10.1016/0010-0285(91)90003-7 2001615

[11] Bock, K., Nicol, J., & Cutting, J. C. (1999). The ties that bind: Creating number agreement in speech. Journal of Memory and language, 40(3), 330–346. 10.1006/jmla.1998.2616

[12] Boroditsky, L., Schmidt, L. A., & Phillips, W. (2003). Sex, syntax, and semantics. In Gentner, D. & Goldin-Meadow, S. (Eds.), Language in mind: Advances in the study of language and thought (pp. 61–79). MIT Press. 10.7551/mitpress/4117.003.0010

[13] Cuetos, F., Glez-Nosti, M., Barbón, A., & Brysbaert, M. (2011). SUBTLEX-ESP: Spanish word frequencies based on film subtitles. Psicológica, 32(2), 133–143.

[14] Davies, M. (2002–) Corpus del Español: Historical/Genres. http://www.corpusdelespanol.org/hist-gen/.

[15] Davies, M. (2016–) Corpus del Español: Web/Dialects. http://www.corpusdelespanol.org/web-dial/

[16] Davies, M. (2018–) Corpus del Español: NOW (2012–2019). http://www.corpusdelespanol.org/now/

[17] Duchon, A., Perea, M., Sebastián-Gallés, N., Martí, A., & Carreiras, M. (2013). EsPal: One-stop shopping for Spanish word properties. Behavior research methods, 45, 1246–1258. 10.3758/s13428-013-0326-1 23468181

[18] Duñabeitia, J. A., Crepaldi, D., Meyer, A. S., New, B., Pliatsikas, C., Smolka, E., & Brysbaert, M. (2018). MultiPic: A standardized set of 750 drawings with norms for six European languages. Quarterly Journal of Experimental Psychology, 71(4), 808–816. 10.1080/17470218.2017.1310261 28326995

[19] Eberhard, K. M. (1997). The marked effect of number on subject–verb agreement. Journal of Memory and Language, 36(2), 147–164. 10.1006/jmla.1996.2484

[20] Eberhard, K. M., Cutting, J. C., & Bock, K. (2005). Making syntax of sense: Number agreement in sentence production. Psychological Review, 112(3), 531–559. 10.1037/0033-295X.112.3.531 16060750

[21] Fábregas, A. (2013). Differential object marking in Spanish: State of the art. Borealis–An International Journal of Hispanic Linguistics, 2(2), 1–80. 10.7557/1.2.2.2603

[22] Franck, J., Vigliocco, G., Antón-Méndez, I., Collina, S., & Frauenfelder, U. H. (2008). The interplay of syntax and form in sentence production: A cross-linguistic study of form effects on agreement. Language and Cognitive Processes, 23(3), 329–374. 10.1080/01690960701467993

[23] Hawkins, D. M. (1980). Identification of outliers. Springer Dordrecht. 10.1007/978-94-015-3994-4

[24] Igoa, J. M., García-Albea, J. E., & Sánchez-Casas, R. (1999). Gender-number dissociations in sentence production in Spanish. Rivista di Linguistica, 11(1), 163–196.

[25] Jescheniak, J. D., Schriefers, H., & Hantsch, A. (2001). Semantic and phonological activation in noun and pronoun production. Journal of Experimental Psychology: Learning, Memory, and Cognition, 27(4), 1058–1078. 10.1037/0278-7393.27.4.1058 11486919

[26] Kandel, M., & Phillips, C. (2022). Number attraction in verb and anaphor production. Journal of Memory and Language, 127, 104370. 10.1016/j.jml.2022.104370

[27] Kandel, M., Wyatt, C. R., & Phillips, C. (2022). Agreement attraction error and timing profiles in continuous speech. Glossa Psycholinguistics, 1(1). 10.5070/G601157

[28] Kandel, M., Wyatt, C. R., & Phillips, C. (2024). Number attraction in pronoun production. Open Mind: Discoveries in Cognitive Science, 8, 1247–1290. 10.1162/opmi_a_00167 39544358 PMC11563651

[29] Levelt, W. J. M. (1989). Speaking: From intention to articulation. MIT Press. 10.7551/mitpress/6393.001.0001

[30] Levelt, W. J., Roelofs, A., & Meyer, A. S. (1999). A theory of lexical access in speech production. Behavioral and brain sciences, 22(1), 1–38. 10.1017/S0140525X99001776 11301520

[31] Lorimor, H., Jackson, C. N., & Foote, R. (2015). How gender affects number: Cue-based retrieval in agreement production. Language, Cognition and Neuroscience, 30(8), 947–957. 10.1080/23273798.2015.1047461

[32] McAuliffe, M., Socolof, M., Mihuc, S., Wagner, M., & Sonderegger, M. (2017). Montreal Forced Aligner: Trainable text-speech alignment using Kaldi. Interspeech, 2017, 498–502. 10.21437/Interspeech.2017-1386

[33] Meyer, A. S., & Bock, K. (1999). Representations and processes in the production of pronouns: Some perspectives from Dutch. Journal of Memory and Language, 41(2), 281–301. 10.1006/jmla.1999.2649

[34] Nozari, N., & Omaki, A. (2022, January 17). An investigation of the dependency of subject-verb agreement on inhibitory control processes in sentence production. 10.31234/osf.io/9pcmg

[35] Prado, M. (1982). El género en español y la teoría de la marcadez. Hispania, 65(2), 258–266. https://www.jstor.org/stable/341541

[36] Prolific (2023). www.prolific.com

[37] R Core Team (2024). R: A language and environment for statistical computing. R Foundation for Statistical Computing, Vienna, Austria. https://www.R-project.org/

[38] Ryskin, R., Stearns, L., Bergen, L., Eddy, M., Fedorenko, E., & Gibson, E. (2021). An ERP index of real-time error correction within a noisy-channel framework of human communication. Neuropsychologia, 158, 107855. 10.1016/j.neuropsychologia.2021.107855 33865848

[39] Schmitt, B. M., Meyer, A. S., & Levelt, W. J. (1999). Lexical access in the production of pronouns. Cognition, 69(3), 313–335. 10.1016/s0010-0277(98)00073-0 10193050

[40] Slevc, L. R., Lane, L. W., & Ferreira, V. S. (2007). Pronoun production: Word or world knowledge. MIT Working papers in Linguistics, 53, 191–203.

[41] Slioussar, N., & Malko, A. (2016). Gender agreement attraction in Russian: Production and comprehension evidence. Frontiers in Psychology, 7, 1651. 10.3389/fpsyg.2016.01651 27867365 PMC5095607

[42] Thornton, R., & MacDonald, M. (2003). Plausibility and grammatical agreement. Journal of Memory and Language, 48(4), 740–759. 10.1016/S0749-596X(03)00003-2

[43] Veenstra, A., Acheson, D., & Meyer, A. (2014). Keeping it simple: Studying grammatical encoding with lexically reduced item sets. Frontiers in Psychology, 18, 783. 10.3389/fpsyg.2014.00783 PMC4103081 25101039

[44] Vigliocco, G. (1996). One or more labels on the bottles? Notional concord in Dutch and French. Language and Cognitive Processes, 11(4), 407–442. 10.1080/016909696387169

[45] Vigliocco, G., Butterworth, B., & Garrett, M. F. (1996). Subject-verb agreement in Spanish and English: Differences in the role of conceptual constraints. Cognition, 61(3), 261–298. 10.1016/S0010-0277(96)00713-5 8990974

[46] Vigliocco, G., Butterworth, B., & Semenza, C. (1995). Constructing subject–verb agreement in speech: The role of semantic and morphological factors. Journal of Memory and Language, 34(2), 186–215. 10.1006/jmla.1995.1009

[47] Vigliocco, G., & Franck, J. (1999). When sex and syntax go hand in hand: Gender agreement in language production. Journal of Memory and Language, 40(4), 455–478. 10.1006/jmla.1998.2624

[48] Vigliocco, G., & Nicol, J. (1998). Separating hierarchical relations and word order in language production: Is proximity concord syntactic or linear? Cognition, 68(1), B13–B29. 10.1016/S0010-0277(98)00041-9 9775519

[49] Von Heusinger, K., & Kaiser, G. A. (2003). The interaction of animacy, definiteness and specificity in Spanish. Proceedings of the Workshop Semantic and Syntactic Aspects of Specificity, Romance Languages, 41–65.

[50] Von Heusinger, K., & Kaiser, G. A. (2007). Differential object marking and the lexical semantics of verbs in Spanish. Proceedings of the Workshop Definiteness, Specificity and Animacy in Ibero-Romance Languages, 85–110.

[51] Zehr, J., & Schwarz, F. (2018). PennController for Internet Based Experiments (IBEX). 10.17605/OSF.IO/MD832

