# Supplementary material for: Number and Grammatical Gender Attraction in Spanish Pronouns: Evidence for a Syntactic Route to Their Features
Source: J Cogn. 2025 Jan 7;8(1):10. doi: 10.5334/joc.416 (PMC11720697; doi:10.5334/joc.416)
Supplement: Supplemental File 1. — Forced-choice study to select the target structures. [file joc-8-1-416-s1.pdf]

Supplemental file 1. Forced-choice study to select the target structures

When selecting the potential adverbial structure to be used in the target sentences in Experiments 1 and 2, there were at least two options that seemed common and frequent in Spanish: a post-attractor segment with the preposition “de” (e.g., “de debajo de él”) or without it (“debajo de él”). A forced-choice task was run via Prolific (www.prolific.com) to check whether there was a preference for either of these structures. Twenty-four adult native Spanish speakers (12 female) with a mean age of 26.7 (SD: 7.7) years participated. They were shown videos similar to those of the experimental trials and were asked to choose one of three options: a sentence with an adverb preceded by the preposition “de”, a sentence with an adverb without the preceding preposition, and a no-preference option (“No tengo preferencia”). The no-preference option was always listed as the third option, while the position of the other two options was counterbalanced across trials.

The stimuli of the forced-choice task were four experimental items randomly selected from Experiment 1. To avoid attraction effects, both nouns matched in number and gender (2 masculine-masculine, 2 feminine-feminine; always singular). To avoid non-words, we replaced the verb “pipear” with a real Spanish verb: “oscurecer” (‘to darken’), which approximately described the content of the video. We counterbalanced the lexical content of the adverb, such that two items appeared with ‘above’ and two with ‘below’.

The results showed a similar preference for the preposition and no-preposition options (Figure S1.1, left panel). Inspection of the results by-participant showed that twelve participants had a preference for prepositional structures, while nine participants had a preference for structures without a preposition (Figure S1.1, right panel). These preferences did not seem to vary depending on the gender of the nouns or the type of adverb (above/below). When asked about the confidence in their answers, 20 out of 24 participants chose a rating of 6 or 7 on a 7-point scale, thus showing clear confidence in their choices. Therefore, given that both the preposition and no-preposition options were preferred similarly, both were allowed as target responses in the experiments. Both structures were introduced in the examples during the familiarization process, and participants were instructed to use the one that sounded more natural to them.

Figure S1.1.

Descriptive summary of preferences in the forced-choice study

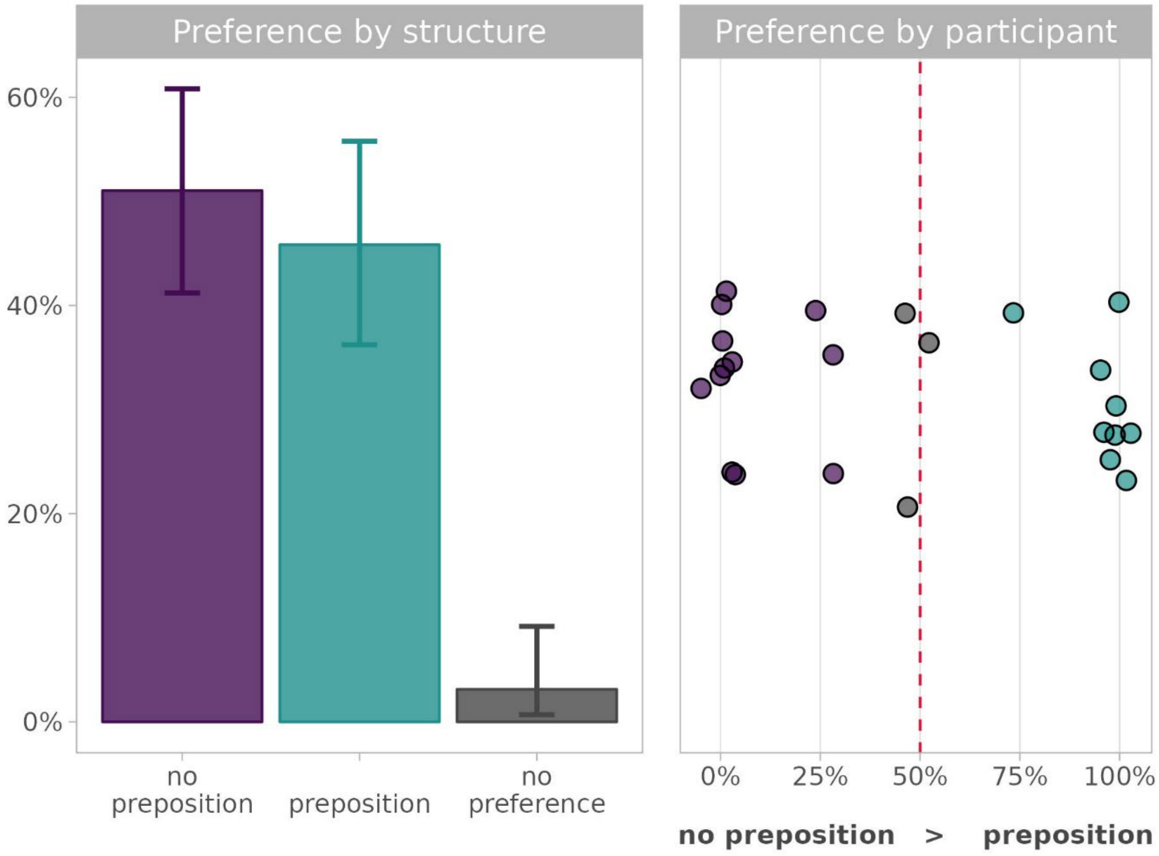

Note. On the left panel, error bars show binomial confidence intervals. On the right panel, each dot represents one participant.
