# Supplementary material for: Number and Grammatical Gender Attraction in Spanish Pronouns: Evidence for a Syntactic Route to Their Features
Source: J Cogn. 2025 Jan 7;8(1):10. doi: 10.5334/joc.416 (PMC11720697; doi:10.5334/joc.416)
Supplement: Supplemental File 2. — Evaluation of the forced aligner. [file joc-8-1-416-s2.pdf]

Supplemental file 2. Evaluation of the forced aligner

We evaluated the performance of the Montreal Forced Aligner with a method commonly used in previous research with forced-aligners (Gonzalez, Grama & Travis 2020; MacKenzie & Turton 2020; McAuliffe et al. 2017). We adapted the method to evaluate the aligner performance at the word — rather than the phoneme — level, since words were the relevant units for our analysis. The procedure was as follows. Two human coders, who were blind to the aligner performance, annotated the boundaries of each word — onsets and offsets — in Praat (Boersma 2001). Their alignments were randomly named H1 (benchmark) and H2 (second human coder) and compared with each other and with those of the forced aligner (FA). The comparison consisted of calculating "boundary displacements", which measured the rate of agreement between H1–H2 (human–human agreement) and H1–FA (human–aligner agreement). We quantified the proportion of boundary displacements that fell within a threshold of 20 ms — a common threshold in previous work (e.g., Cosi, Falavigna & Omologo 1991; Goldman 2011; Hosom 2009; Raymond et al. 2002; Wilbanks 2015). For example, a human–aligner agreement rate of 80% meant that 80% of the boundaries from the forced aligner fell within 20 ms of the boundaries from the human benchmark.

The use of the boundary displacement method allowed us to quantify how the agreement rates in our experiments compared to those of previous studies with forced aligners and a 20 ms threshold, as reported in a recent review article (Gonzalez, Grama & Travis 2020). For human–aligner (HA) comparisons, previous studies have reported agreement rates ranging from 56% (Cosi, Falavigna & Omologo 1991) to 93% (Hosom 2009). For human–human (HH) comparisons, previous studies have reported agreement rates between 79% (McAuliffe et al. 2017; Raymond et al. 2002; Goldman 2011) and 93% (Hosom 2009). We used those ranges as reference points, but we expected to obtain lower agreement rates for several reasons. First, the use of web-based data collection meant that the quality of our recordings was necessarily lower than those of previous studies, usually performed in a lab setting. Second, previous studies typically featured a small number of speakers, in contrast with our experiments, which featured 47–72 speakers coming from different parts of Spain. Due to the lack of previous psycholinguistic sentence production studies using the boundary displacement method, we can't assess the representativeness of our agreement rates. Nevertheless, we provide them below because we believe they are an important step to improve methodological robustness in production experiments that use forced aligners.

Experiment 1

The aligner performance in Experiment 1 was evaluated with a sample of 188 trials (4.7% of the latency data), which were equally distributed across participants and experimental conditions. For the HA comparison, the agreement rate across all sentence segments was 60%, thus falling within the range observed in previous studies (56–93%). For the HH comparison, the agreement rate across all segments was 65.4%, which was lower than in previous studies (79–93%). The lower agreement rate between our two human coders might be due to the fact that they spoke different varieties of Spanish (Peninsular Spanish vs. Rioplatense Spanish).

In a second step, we focused on the segments that were directly relevant to define the critical post-attractor segment used in our latency analyses: the attractor and the pronoun offset. For the attractor offset, the HA agreement rate was 54.8% and the HH rate was 70.2%. For the pronoun offset, the HA agreement rate was 37.2% and the HH rate was 47.9%. This shows that there was relatively low agreement — between humans and between the human and the aligner — about the pronoun offset. To ensure that this variability did not have a systematic influence in the between-condition comparisons in our analyses, we plotted the distribution of the absolute boundary displacements for the pronoun offset (Figure S2.1). The figure did not suggest any evidence of a systematic bias. For all conditions, the boundary displacements were almost similarly distributed around zero (perfect alignment), both for human–human and human–aligner comparisons. Therefore, while there was little agreement about the exact location of the pronoun offset, we deem it unlikely that this had a confounding effect in the between-condition comparisons.

Figure S2.1.

Distribution of boundary displacements for the pronoun offset in Experiment 1

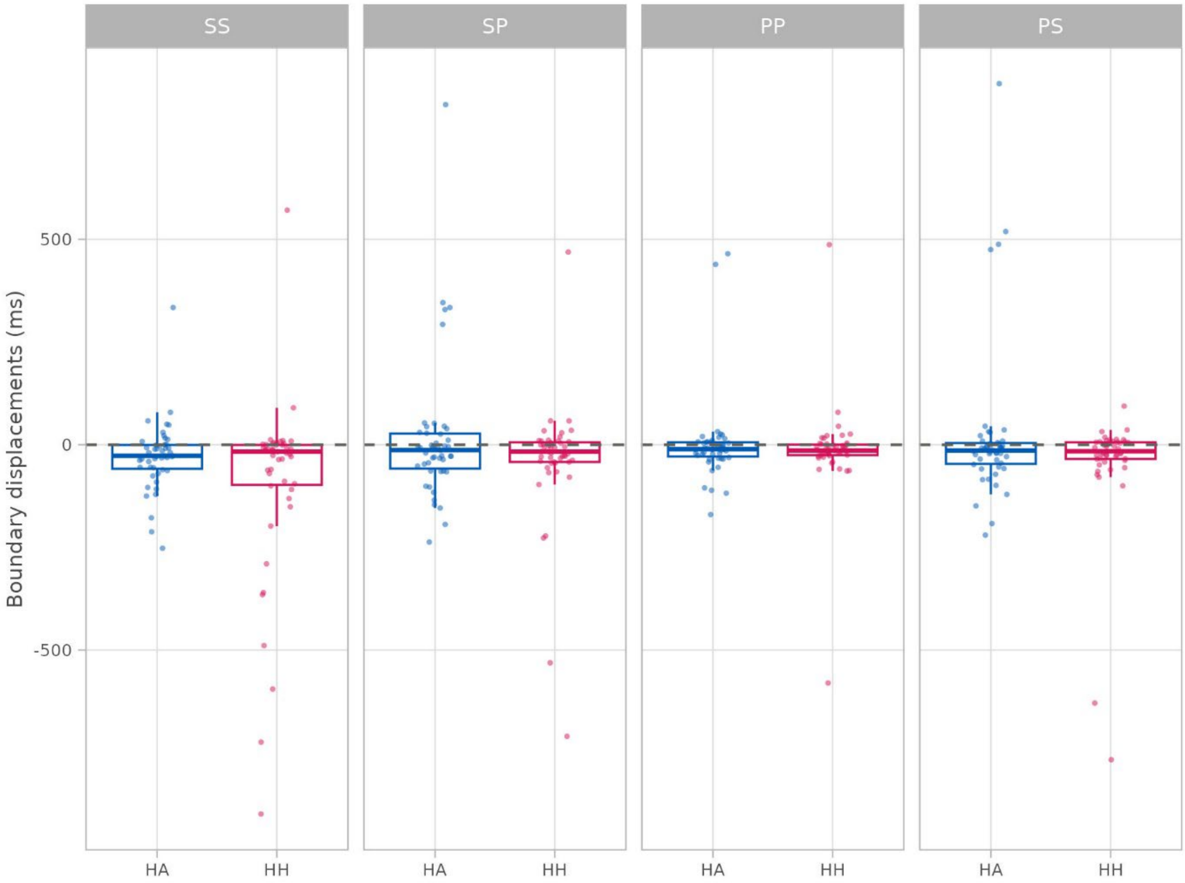

Note. Points show by-participant averages. The dashed horizontal line at 0 represents full agreement between the two aligners — i.e., a 0 ms boundary displacement. A positive/negative value shows a positive/negative deviation of the comparison alignment from the human benchmark. Abbreviations: HA = human–aligner comparison, HH = human–human comparison, SS = singular antecedent, singular attractor, SP = singular antecedent, plural attractor, PP = plural antecedent, plural attractor, PS = plural antecedent, singular attractor.

Experiment 2

The aligner performance in Experiment 2 was evaluated with a sample of 288 trials (4.4% of the latency data), which were equally distributed across participants and experimental conditions. For the human–aligner comparison, the agreement rate across all sentence segments was 57.9%, thus falling within the range observed in previous studies (56–93%). For the human–human comparison, the agreement rate across all segments was 73.0%, which was lower than in previous studies (79–93%). As noted for Experiment 1, the lower agreement rate between our two human coders might be due to the fact that they spoke different varieties of Spanish (Peninsular Spanish vs. Rioplatense Spanish).

In a second step, we focused on the segments that were directly relevant to define the critical post-attractor segment: the attractor and the pronoun offset. For the attractor offset,

the HA agreement rate was 69.1% and the HH rate was 69.4%. For the pronoun offset, the HA agreement rate was 47.2% and the HH rate was 59.7%. These agreement rates were higher than in Experiment 1, but there was still less agreement about the pronoun offset as compared to other boundaries. To ensure that this variability did not have a systematic influence in the between-condition comparisons, we plotted the distribution of the absolute boundary displacements for the pronoun offset (Figure S2.2). The figure did not suggest any evidence of a systematic bias. For all conditions, the boundary displacements were similarly distributed around zero (perfect alignment), both for the human–human and human–aligner comparisons. The only condition where the two alignments differed more was FF: the human aligner disagreed with the human benchmark by setting a later boundary for the end of the pronoun, while the forced-aligner was less consistent. In general, while there was little agreement about the exact location of the pronoun offset, we deem it unlikely that this had a confounding effect in the between-condition comparisons.

Figure S2.2.

Distribution of boundary displacements for the pronoun offset in Experiment 2

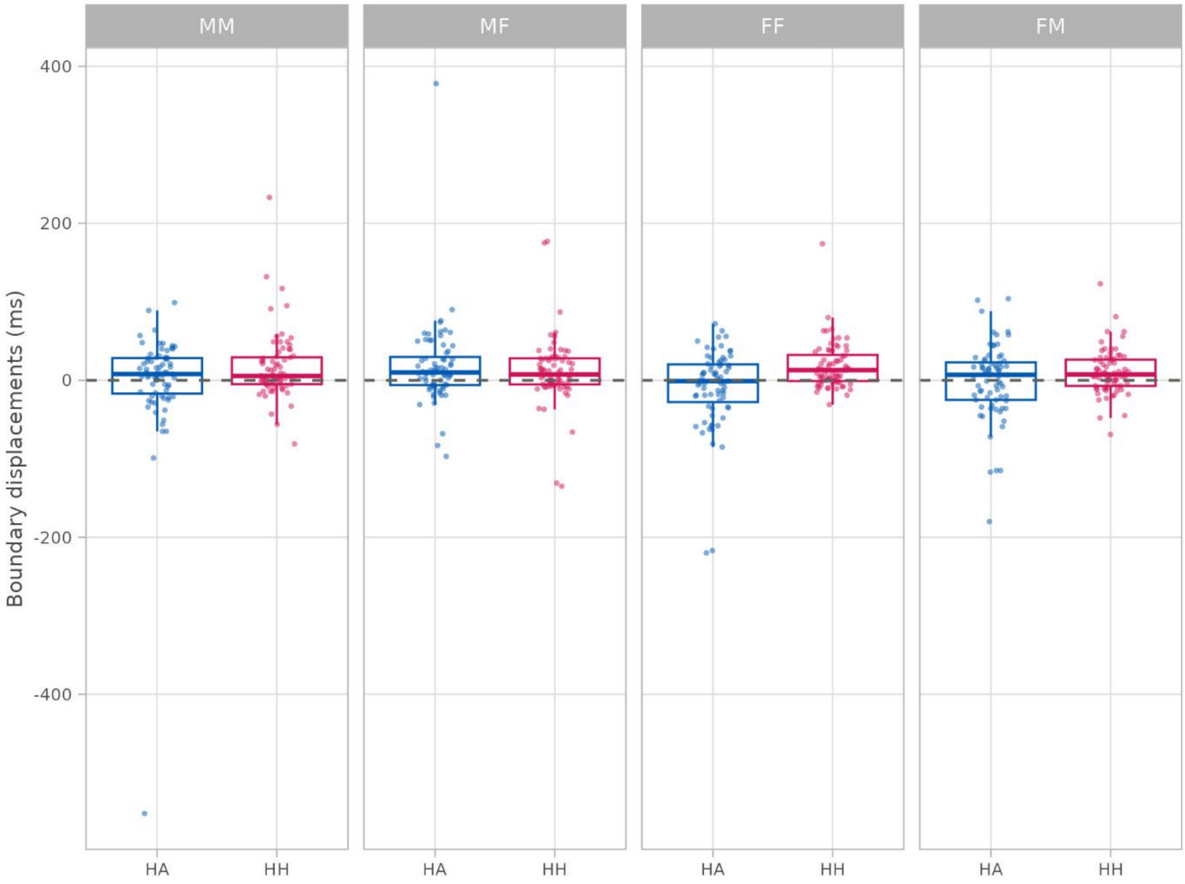

Note. Points show by-participant averages. The dashed horizontal line at 0 represents full agreement between the two aligners. A positive/negative value shows a positive/negative deviation of the comparison alignment from the human benchmark. Abbreviations: HA = human-aligner comparison alignment, HH = human-human comparison alignment, MM = masculine antecedent, masculine attractor, MF = masculine antecedent, feminine attractor, FF = feminine antecedent, feminine attractor, FM = feminine antecedent, masculine attractor.

References

Boersma, P. (2001). Praat, a system for doing phonetics by computer. *Glot International*, *5*(9), 341–345.

Cosi, P., Falavigna, D., & Omologo, M. (1991). A preliminary statistical evaluation of manual and automatic segmentation discrepancies. *Proceedings of the Second European Conference on Speech Communication and Technology (Eurospeech 1991)*, 693–696. https://doi.org/10.21437/Eurospeech.1991-183

Goldman, J.-P. (2011). Easyalign: An automatic phonetic alignment tool under Praat. *Proceedings of the 12th Conference of the International Speech Communication Association (Interspeech 2011)*, 3233–3236. https://doi.org/10.21437/Interspeech.2011-815

Gonzalez, S., Grama, J., & Travis, C. E. (2020). Comparing the performance of forced aligners used in sociophonetic research. *Linguistics Vanguard*, *6*(1), 20190058. https://doi.org/10.1515/lingvan-2019-0058

Hosom, J.-P. (2009). Speaker-independent phoneme alignment using transition-dependent states. *Speech Communication*, *51*(4), 352–368. https://doi.org/10.1016/j.specom.2008.11.003

MacKenzie, L., & Turton, D. (2020). Assessing the accuracy of existing forced alignment software on varieties of British English. *Linguistics Vanguard*, *6*(s1), 20180061. https://doi.org/10.1515/lingvan-2018-0061

McAuliffe, M., Socolof, M., Mihuc, S., Wagner, M., & Sonderegger, M. (2017). Montreal Forced Aligner: Trainable text-speech alignment using Kaldi. *Interspeech 2017*, 498–502. https://doi.org/10.21437/Interspeech.2017-1386

Raymond, W. D., Pitt, M., Johnson, K., Hume, E., Makashay, M., Dautricourt, R., & Hilts, C. (2002). An analysis of transcription consistency in spontaneous speech from the buckeye corpus. *Proceedings of the 7th International Conference on Spoken Language Processing (ICSLP 2002)*, 1125–1128. https://doi.org/10.21437/ICSLP.2002-371

Wilbanks, E. (2015, October). *The development of FASE: Forced Alignment System for*

Español and implications for sociolinguistic methodologies. Talk given at New Ways of Analyzing Variation 44, Toronto, Canada.
