# Supplementary material for: Number and Grammatical Gender Attraction in Spanish Pronouns: Evidence for a Syntactic Route to Their Features
Source: J Cogn. 2025 Jan 7;8(1):10. doi: 10.5334/joc.416 (PMC11720697; doi:10.5334/joc.416)
Supplement: Supplemental File 3. — Supplementary analyses. [file joc-8-1-416-s3.pdf]

Supplemental file 3. Supplementary analyses

Error rates

Table S3.1

Error rates by condition in Experiment 1

Condition	Number of errors	Number of trials	Percentage (%)
SS	7	1185	0.591
SP	52	1142	4.553
PP	16	1128	1.418
PS	44	1124	3.915

Note. Abbreviations: SS = singular antecedent, singular attractor, SP = singular antecedent, plural attractor, PP = plural antecedent, plural attractor, PS = plural antecedent, singular attractor.

Table S3.2

Error rates by condition in Experiment 2

Condition	Number of errors	Number of trials	Percentage (%)
MM	48	1879	2.555
MF	89	1903	4.677
FF	81	1920	4.219
FM	147	1888	7.786

Note. Abbreviations: MM = masculine antecedent, masculine attractor, MF = masculine antecedent, feminine attractor, FF = feminine antecedent, feminine attractor, FM = feminine antecedent, masculine attractor.

Exploratory analysis: Comparison of number and gender attraction

Table S3.3

Output of the error analysis model

Coefficient	Estimate	Standard error	z-value	p-value
Intercept (grand mean)	–3.837	0.128	–30.085	< 0.001
Trial Order	–0.000	0.001	–0.027	0.978
Experiment	0.898	0.239	3.764	< 0.001
Match	1.134	0.151	7.534	< 0.001
Experiment × Match	–0.793	0.300	–2.643	0.008

Note. Model formula: Error ~ Trial Order + Experiment * Match + (1 + Match || Participant) + (1 + Match || Item). A positive coefficient for Experiment reflects more errors in experiment 2 than experiment 1. A positive coefficient for Match reflects a greater likelihood of errors for mismatch than match conditions. The double bars in the model formula represent the removal of the correlation between random slopes and intercepts.

Table S3.4

Output of the latency analysis model

Coefficient	Estimate	Standard error	t-value	p-value
Intercept (grand mean)	6.665	0.009	765.159	< 0.001
Trial Order	–0.000	0.000	–3.858	< 0.001
Syllable Count	0.178	0.003	55.604	< 0.001
Experiment	0.017	0.017	0.975	0.331
Match	0.009	0.003	2.797	0.006
Experiment × Match	–0.014	0.006	–2.223	0.028

Note. Model formula: log(Duration) ~ Trial Order + Syllable Count + Experiment * Match + (1 + Match | Participant) + (1 + Match | Item). A positive coefficient for Experiment reflects longer durations in experiment 2 than experiment 1. A positive coefficient for Match reflects longer durations for mismatch than match conditions.
